# Editorial: Face Perception across the Life-Span

**DOI:** 10.3389/fpsyg.2016.01338

**Published:** 2016-08-31

**Authors:** Bozana Meinhardt-Injac, Andrea Hildebrandt

**Affiliations:** ^1^Department of Psychology, Johannes Gutenberg-Universität MainzMainz, Germany; ^2^Department of Psychology, Ernst-Moritz-Arndt-Universität GreifswaldGreifswald, Germany

**Keywords:** face perception, emotion perception, visual processing, life-span development, individual differences

Faces convey information that is of great importance for humans as social beings. The ability to process information from faces undergoes significant changes across the life-span (e.g., Germine et al., 2011), and shows considerable individual differences (e.g., Wilhelm et al., 2010). Average developmental trends and changes of individual differences in face perception across the life-span arise from multiple components. These include sensory (e.g., holistic, configural, and feature based perception), cognitive (e.g., memory, processing speed, attentional control) and emotion related (e.g., identifying facial expression) processing domains. Because our understanding of rather isolated functional domains involved in face perception was growing during the last decades of research, for the present research topic we called for multi-component approaches toward an integrated view on facial information processing. We anticipated that such an approach may help to better describe how the multifaceted facial processing ability is composed and how the components relate to each other. Thus, we aimed to bring together a collection of papers to provide a shot of the current state of the art in developmental research that illustrate actual trends at theorizing and investigating the components of face processing in the context of related abilities. We were open for submissions focusing on average life-span trends, on changes of individual differences, or both.

Nineteen successfully published submissions contributed to this aim. Their findings suggest that faces are a special object category in many respects. In the *Editorial*, we aim at an integrated view of these contributions. Several papers link findings on different facial information processing abilities, and illuminate their relationships with other cognitive and socio-emotional ability domains in age heterogeneous samples. We will first give an overview of the papers published in the research topic, integrate their findings and derive conclusions for future life-span research on multiple components of face perception.

## Face perception: where development and aging meet

Several regions of the human brain including the amygdala, the superior temporal sulcus (STS) and the fusiform face area (FFA) are tuned to different kinds of facial information (i.e., featural and configural; Golarai et al.). There is evidence on the heritability of face processing behavior (e.g., Wilmer et al., 2010) that implies the neural face-system to be inborn, at least to some extent. This system is responsive to faces or face-like configurations (e.g., up-down asymmetry) already at birth, and becomes more and more tuned to human faces during development as a function of visual experience (Simon and Di Giorgio). Cognitive specialization at processing faces occurs in the first months and years of life and still continues across childhood and adolescence. For example, protracted development may be reflected by an increased sensitivity to second-order configural information that refers to the representation of spatial relations between facial features (e.g., inter-eye distances—Meinhardt-Injac et al.; Joseph, et al.). An impaired processing of the second-order configuration in faces is present in the Williams syndrome already in infancy, while processing of facial features seems to be unaffected (souza et al.). These findings may be interpreted as reflecting the particular role of adjustment of the human face-system to second-order configurations. In childhood and adolescence, however, there are also some functional commonalities in face and object processing that are typically not observed in adulthood (Joseph et al.; Jüttner et al.). Accordingly, the development of face perception could be understood as a process where domain-general and domain-specific mechanisms dissociate across childhood and adolescence as a result of increasing face-related expertise (e.g., Wang et al., 2016). The development of domain-specific processes can proceed at different rates for different modules of face processing (e.g., Weigelt et al., 2014).

The sensitivity to configural information in faces shows not only protracted development, but also an early decline, starting at about 50 years of age (Meinhardt-Injac et al.; see also Chaby et al., 2001, 2011). In a comprehensive review, Boutet et al. conclude that impairment in the processing of configural information may be one of the major factors of age-related decline in face processing ability. Other possible factors affecting face processing in older age are the decline in basic sensory abilities and faded context recollection. In line with this argument, Olderbak et al. demonstrate that common variance shared by vision, fluid cognitive ability, and immediate and delayed memory predict some but not all age-related variance in face perception and face memory. Taken together, these studies suggest domain-specific aging of the face processing system that cannot be accounted for by domain-general aging processes (e.g., Hildebrandt et al., 2011).

Not only the age of the perceiver affects face perception, but also face-age plays an important role in processing facial information. Face stimuli of different ages seem to trigger different perceptual mechanisms, where categorization of older-face depend more strongly on local texture-based information than it is the case for young faces (Komes et al.). These face-age effects are not only salient in perceptual mechanisms, but also in memory. Fodarella et al. demonstrates an advantage in naming (i.e., memory) for older faces in older subjects (i.e., Own Age Bias, OAB) in facial-composite construction.

The impact of perceptual expertise with different stimulus domains (e.g., language, non-face visual objects) on higher order cognition has been well-documented. Bulf et al. extended available knowledge to the social domain by showing how perceptual expertise with upright versus inverted faces affects rule learning in young infants. On the other hand, top-down influences on face perception are becoming more and more recognized. Luo et al. reviewed literature describing the neural systems and hormones involved in perceiving the cuteness of infant faces. The identified broad neural circuitry, comprising face and emotion processing, as well as reward and attachment related brain regions, demonstrate top-down influences on person perception and, specifically, on the perception of attractiveness.

## Emotion perception: from labeling unimodal stimuli to evaluating facial expressions

The development of the ability to differentiate facial expressions has been extensively studied in the past. However, normative data on developmental trajectories is surprisingly scarce. Lawrence et al. provide comprehensive cross-sectional data that allow estimating the developmental trajectory of facial emotion recognition between the ages of 6–16 years. Particular for this study is that a standardized and unitary emotion labeling method was used across the whole age range. Children and adolescents labeled emotions expressed by adults from the Ekman-Friesen Pictures of Facial Affect. These emotion recognition data, controlled for IQ, allow differential comparisons related to basic emotion categories, showing that sadness and anger expression recognition is almost at maturity at mid childhood age (about 6 years), whereas linear increase characterizes happiness, surprise, disgust, and fear recognition across the observed age range.

Decline of emotion recognition in older age cannot be studied without considering its interplay with cognition and emotion regulation. Research described by Di Domenico et al. using a dynamic facial expression recognition and a subsequent intensity evaluation task suggests a positivity bias during online emotion identification in older as compared with younger adults. This phenomenon may be driven by well-documented emotion regulation priorities in older age. When tested in isolation, older adults consistently prove to be impaired in recognizing emotions in several modalities. This has been observed for both faces and voices. However, research on the use of cross-modal integration for emotion recognition that argues for an advantage in older ages still needs scientific attention. Chaby et al. supply data comparing younger and older adults indicating similar benefit provided by multimodal information in older and younger ad Souza, ults.

In everyday affective communication humans often neutralize, mask and simulate emotional expressions. Thus, evaluating the authenticity of facial expressions is (above identification of basic emotions) a crucial ability for mastering social interactions. Dawel et al. describe data suggesting authenticity recognition to be characterized by late maturity. Whereas children were less skilled at identifying genuine smiles as compared with young adults, they performed above chance levels in happiness authenticity recognition. However, children could not differentiate genuine sadness and fear expressions from faked ones. Adults also failed to correctly identify the authenticity of fearful facial expressions.

## Mutual influence of emotion and appearance on processing invariant and variant facial information

Neuro-cognitive models on person perception usually differentiate the processing route of invariant (identity related) and variant (emotion, face speech, gaze direction) facial information. However, to date mutual influences between the two routes are well-recognized. The research topic includes three endeavors to this topic. First, every face tends to express an emotion expression even in a neutral state. These characteristic, so called baseline expressions of faces have an influence on person perception in adult receiver. For example, adults perceive faces displaying anger as being more masculine as compared with faces displaying different emotions. Bayet et al. show the anger bias toward male categorization to be present already in children as young as 5–6 years. They also report computational simulations of gender categorization, which together with the developmental data indeed do not refute or confirm the mechanism behind the male-bias associated with anger expressions, but emphasize the role of experience-based perceptual inferences and belief-based inferences (stereotype) to this phenomenon. Second, it is conceivable that facial expressivity leaves long-term marks on faces across the life-span and these will influence person and affect perception from a given face. Adams et al. describe data supporting this assumption and show that expressive ratings of neutral facial displays predicted self-reported positive affect of elderly women. Third, not only expressions influence person perception, but also facial appearance has an impact on emotion recognition. Aspects of this phenomenon are illustrated by Freudenberg et al., who showed that misattributions of emotions to elderly faces impair facial emotion processing at several levels of performance.

## Theoretical integration

Functional models (e.g., Haxby et al., 2000; Young and Bruce, 2011) postulated a hierarchical structure of facial information processing. Above identity processing—thus of invariant facial information—these models allow predictions about how the system deals with variable information provided by faces, including emotional expressions and gaze direction. Socially relevant information that can be further derived from the invariant face structure are age, gender, judgments about attractiveness etc. Early theorizing assumed independent streams of identity versus expression related information. This assumption was recently modified in favor of partial dependence views (e.g., Calder, 2011). However, it is not yet fully clarified how these systems interact. This research topic contributes with some further knowledge about this interaction and aims to trigger future developmental research in this area.

## Conclusion

The research topic provides evidence on developmental trajectories and aging effects of processing identity and expression related information from faces. The life-span development of these abilities have been studied in their interplay with components of these abilities and a series of higher-order cognitive functions. Three further papers described research that has been dedicated to studying mutual influences of emotion and appearance on processing invariant and variant facial information. While the efficiency of face processing is clearly affected by age, we need extensive research to reveal and to separate effects that are domain-specific from those that are possibly domain-general. Moreover, possible cohort effects not only in cognitive, but also socio-emotional domains need to be controlled for in future research.

## Author contributions

All authors listed, have made substantial, direct and intellectual contribution to the work, and approved it for publication.

### Conflict of interest statement

The authors declare that the research was conducted in the absence of any commercial or financial relationships that could be construed as a potential conflict of interest.
